# Autonomous Obstacle Crossing Strategies for the Hybrid Wheeled-Legged Robot Centauro

**DOI:** 10.3389/frobt.2021.721001

**Published:** 2021-11-19

**Authors:** Alessio De Luca, Luca Muratore, Vignesh Sushrutha Raghavan, Davide Antonucci, Nikolaos G. Tsagarakis

**Affiliations:** ^1^ Humanoids and Human Centered Mechatronics Research Line, Istituto Italiano di Technologia, Genoa, Italy; ^2^ Università di Pisa, Pisa, Italy

**Keywords:** hybrid wheeled-legged planning, legged robots, legged locomotion, robot control, field robotics, perception, trajectory generation

## Abstract

The development of autonomous legged/wheeled robots with the ability to navigate and execute tasks in unstructured environments is a well-known research challenge. In this work we introduce a methodology that permits a hybrid legged/wheeled platform to realize terrain traversing functionalities that are adaptable, extendable and can be autonomously selected and regulated based on the geometry of the perceived ground and associated obstacles. The proposed methodology makes use of a set of terrain traversing primitive behaviors that are used to perform driving, stepping on, down and over and can be adapted, based on the ground and obstacle geometry and dimensions. The terrain geometrical properties are first obtained by a perception module, which makes use of point cloud data coming from the LiDAR sensor to segment the terrain in front of the robot, identifying possible gaps or obstacles on the ground. Using these parameters the selection and adaption of the most appropriate traversing behavior is made in an autonomous manner. Traversing behaviors can be also serialized in a different order to synthesise more complex terrain crossing plans over paths of diverse geometry. Furthermore, the proposed methodology is easily extendable by incorporating additional primitive traversing behaviors into the robot mobility framework and in such a way more complex terrain negotiation capabilities can be eventually realized in an add-on fashion. The pipeline of the above methodology was initially implemented and validated on a Gazebo simulation environment. It was then ported and verified on the CENTAURO robot enabling the robot to successfully negotiate terrains of diverse geometry and size using the terrain traversing primitives.

## 1 Introduction

During the past 2 decades there was an increasing effort in developing robots for addressing challenges in difficult and unstructured environments such as those resulted after physical or man-made catastrophes like earthquakes, nuclear accidents and tsunami. These developments were motivated from the fact that these disaster conditions are highly unsafe for the human emergency responders. In fact, the aforementioned scenarios can put at the risk the health of a person with the presence of radiations, toxic contamination or collapsing structures. To avoid negotiating the health of the emergency responders working under these circumstances, extensive mission training/preparation, attention and planning before entering in a hazardous area is needed to be carried out under the very restricted time constraints of the first response procedures. Unfortunately, even in the case of good awareness of the expected conditions in the critical space, the risks for the operators still remains substantially significant when entering such unstructured and unpredictable environments. For these reasons the robotics community started to develop robotic technologies that can demonstrate the ability of carrying out tasks autonomously or semi-autonomously, keeping the human operators safe.

In particular, to substitute effectively a human responder and operate within a challenging environment in disaster response applications, a robot has to be able to perform a variety of tasks interacting with the entities of the environment, negotiating cluttered ground with obstacles of different shape and dimension, making a way to the final goal assigned.

This highlights the need for a mobile robot (wheeled, legged or hybrid) to be capable of navigating across the environment, identifying possible obstacles and terrain features and negotiating them effectively using a variety of traversing skills to reach the target location.

To achieve such autonomous functionalities, perception driven reasoning about the environment features, geometries and conditions, together with perception driven planning are two of the most fundamental skills needed to be endowed in the emerging robotic systems targeting to operate in such challenging workspaces. Towards this direction, in this work we present an obstacle crossing framework that allows to demonstrate terrain negotiation skills that are adaptable, extendable and can be selected and combined in an autonomous manner based on the features and geometries of the terrain surface as identified by the robot perception system. The main features and contributions of our terrain crossing framework include:

• use of a set of terrain traversing template primitives that are employed to perform driving, support polygon shaping, stepping on, down and over terrain surfaces and obstacles,
• extraction of the terrain geometrical features and obstacles through point cloud data processing and terrain segmentation, identifying possible gaps or obstacles on the ground and their geometrical parameters,
• autonomous selection and serialization of the most appropriate traversing template primitives given the geometrical parameters of the terrain features and obstacle perceived in front of the robot,
• on the fly regulation of the parameters of the terrain traversing template primitives based on the obstacle dimensions,
• and finally a fully extendable architecture that permits to incorporate additional primitive traversing behaviors into the robot mobility framework and in such a way more rich terrain negotiation capabilities can be eventually realized in an add-on fashion.

In more details the robot is able to automatically acquire a representation of the environment, extract the needed information from the obstacles and plan a feasible path that allows it to reach the desired target location regulating and concatenating the available primitives. The human operator enters the loop exclusively to send the target location that the robot has to reach.

The proposed mobility framework is implemented and validated both in simulation and experimentally on the CENTAURO hybrid mobility manipulation robot (Kashiri et al., 2019) endowing the platform with the ability to plan and mix wheeled and stepping actions in fully autonomous manner effectively negotiating obstacles on the ground using the selected, according to the plan, traversing template primitive actions.

The paper is organized as follows: in Section 2 we will briefly analyze some related works in terms of robots for disaster scenarios. Section 3 presents the proposed autonomous obstacle crossing framework. In Section 4 the experimental results are shown and discussed. Finally in Section 5 we outline the conclusion.

## 2 Related Work

One of the high level challenges that any mobile robot experiences when it needs to operate within an unstructured environment, is to be able to demonstrate effective navigation and mobility performance that permits it to deal with the terrain uncertainties and unknown setting of the unstructured environment in general.

In order to achieve this, the robot needs to build a representation of the environment and localize itself in it, updating the map based on the new information coming from the sensory system installed. This problem has been widely addressed in the literature and different methods exist. In Mastalli et al. (2020) and Fankhauser et al. (2018) are proposed methods that work with a 2.5D elevation map, implementing a locomotion framework for quadruped robots. On the other side there are also solutions that use a point cloud to represent the surrounding area and extract the needed information through segmentation. The segmented elements are used to understand the scene composition and then define the desired behaviour as in Oßwald et al. (2011) where a humanoid robot segments the horizontal planes of stairs for a climbing task or more in general as in Wang et al. (2020) where a point cloud representation is used for navigation.

In this work, we decided to use a method based on the segmentation of planes in a point cloud in order be able to segment the needed elements and extract the relevant information more precisely. This would be harder to extract from a 2.5D elevation map due also to the application of smooth filters that degrade the accuracy. Moreover the point cloud can be used for object detection in future developments.

Focusing now on the locomotion aspects, mobile robots can be classified as wheeled, tracked, legged or hybrid. Wheeled and tracked mobility has been widely explored in mobile robots for diverse applications ranging from space exploration rovers (Tunstel et al., 2005) to home cleaning platforms (Tribelhorn and Dodds, 2007). Generally they have less problems of equilibrium but they are limited to drive in flat or low unevenness terrains mostly. To overcome this limitation and enable mobility over rough grounds, legged robots were introduced. Their design generally includes two or more legs enabling them to traverse the environment via stepping. Despite the fact that stepping poses more equilibrium challenges, legged platforms are able to move inside cluttered and degraded environments, gaining the ability to negotiate different kind of obstacles and ground challenges. Examples of legged robots include several humanoid platforms (Radford et al., 2015; Tsagarakis et al., 2017), but also quadruped like HyQ (Semini et al., 2011), ANYmal (Fankhauser and Hutter, 2018) and the Boston Dynamics’ SpotMini.^1^

Hybrid robots target to combine the benefits of both wheeled and legged mobility. They are generally made by legs ending in wheels permitting them to drive on flat surfaces, which is faster, safer and better from an energy consumption point of view. At the same time the legged articulation allows them to also perform stepping and crossing of obstacles adapting completely to the terrain nature and imposed challenges. This makes hybrid robots potentially more effective combining the flexibility of legged mobility with the efficiency of wheels when the terrain is appropriate. Some examples of hybrid mobility robots include Momaro (Schwarz et al., 2016b), CENTAURO (Kashiri et al., 2016), and RoboSimian (Hebert et al., 2015) platforms. In addition, it is worth considering the effort applied by the ANYmal team with the transformation of their legged robot into a hybrid one, attaching wheels to the legs and developing a whole-body motion control and planning framework for hybrid locomotion (Bjelonic et al., 2019; Bjelonic et al., 2020).

Another important aspect to take into account is the strategy used to control a robot operating in an unstructured and remote environment (Atkeson et al., 2016). The first approach that is generally implemented, is through direct teleoperation where a human operator manually controls the robot movements *via* a number of interfaces (as in Hedayati et al., 2018; Bandala et al., 2019). This approach may be easier to realize but it requires to provide effective teleoperation interfaces, train the operators and negotiate potential issues due to delays and degraded communication. Moreover this approach poses a high cognitive load on the human operator especially when he/she has to deal with high complex, number of Degrees of Freedom (DoFs) robots.

To assist the human operator and improve the efficiency in the execution of the tasks, the work in Katyal et al. (2014) incorporate to the teleoperation framework semi-autonomous capabilities based on the task required. Similarly in Klamt et al. (2018) and Klamt et al. (2020), semi-autonomous capabilities were introduced to help the operator in complex tasks like grasping an object. Despite the fact that a semi-autonomous behaviour hides the complexity of some actions, there is still strong dependency on the human operator. To bypass this, the research effort started to concentrate on the development of robots that can demonstrate even higher level of autonomy (Wong et al., 2018), permitting the robots to accomplish tasks without the need of continuous input from the operator. This approach requires the implementation of robust autonomous skills since the robot has to complete the desired tasks without any or with minor human intervention. This can significantly reduce the execution time of the mission requiring the human to enter in the loop for high-level control or for monitoring the execution of the tasks. Concerning the mobility control in particular, the enhancement of autonomous navigation and obstacle crossing skills can significantly increase the potential of mobile robots operating in unstructured and remote environments.

When taking about autonomous navigation, the majority of the work is related to the field of autonomous vehicles as in Abdulmajeed and Mansoor (2014) and Zhiwei et al. (2015), where autonomous mobile platforms use one (or generally more) LiDAR to discover and avoid obstacles. These robotic platforms nowadays are widely used in warehouses or other places but, despite the nice results achieved, wheeled robots are limited for the objective we would like to achieve. Of course, in the last years, a lot of effort was spent also on more complex robotic systems. A simple approach was presented in Rohmer et al. (2008), where the team proposed an Action Planner based on an elevation map for lunar exploration with a hybrid robot. Here the minimal path is extracted and then transitions between driving and stepping mode are identified. In the following years, more elaborated solutions have been proposed.

In Schwarz et al. (2016a) is presented a framework for semi-autonomous locomotion and manipulation with the hybrid robot Momaro, where the operator controlled the movements of the robot, but here only driving and manipulation were considered. This locomotion framework was then extended in Klamt and Behnke (2017), incorporating the robot orientation and a hierarchical step planner, employing an action set defined on the basis of the kinematic capabilities of the robot, but also in this case the framework was not fully autonomous. Similar results can be observed with the wheeled-legged robot Pholus. In Sun et al. (2020), a control framework is proposed for hybrid locomotion built on a hierarchical structure based on: hybrid footstep placement planning, Center of Mass trajectory optimization and whole-body control. In particular, the foot placement planning is executed considering a set of motion modalities defined: driving, walking or, more generally, hybrid modes. This was tested with simple objects, without showing fully autonomous capabilities, in fact they plan to introduce reinforcement learning to determine the motion modes to be used. Few months later, in Haddeler et al. (2020), the team proposed also a motion planning framework for boundary exploration with the possibility to switch between driving and stepping based on the needs. In these experiment the team used a pre-computed map, without evaluating the new data online, due to hardware-limitations. In this work we tackle the problem of autonomous obstacle crossing using the hybrid mobility CENTAURO robot (Kashiri et al., 2016). We introduced a hybrid mobility planner driven by the robot perception, which enables CENTAURO to cross and avoid obstacles on the ground in a complete autonomous fashion starting from an empty map of the environment that is built at run-time. The proposed hybrid mobility planner leverages on a number of terrain traversing primitives that can be modulated, combined and serialized to generate hybrid mobility plans that can then be autonomously executed by CENTAURO. The operator is required to provide the final destination pose to reach and to supervise the execution, which is then autonomously planned and executed. The details of the proposed hybrid mobility planner are introduced in the next sections.

## 3 Obstacle Crossing Framework

**FIGURE 1:**
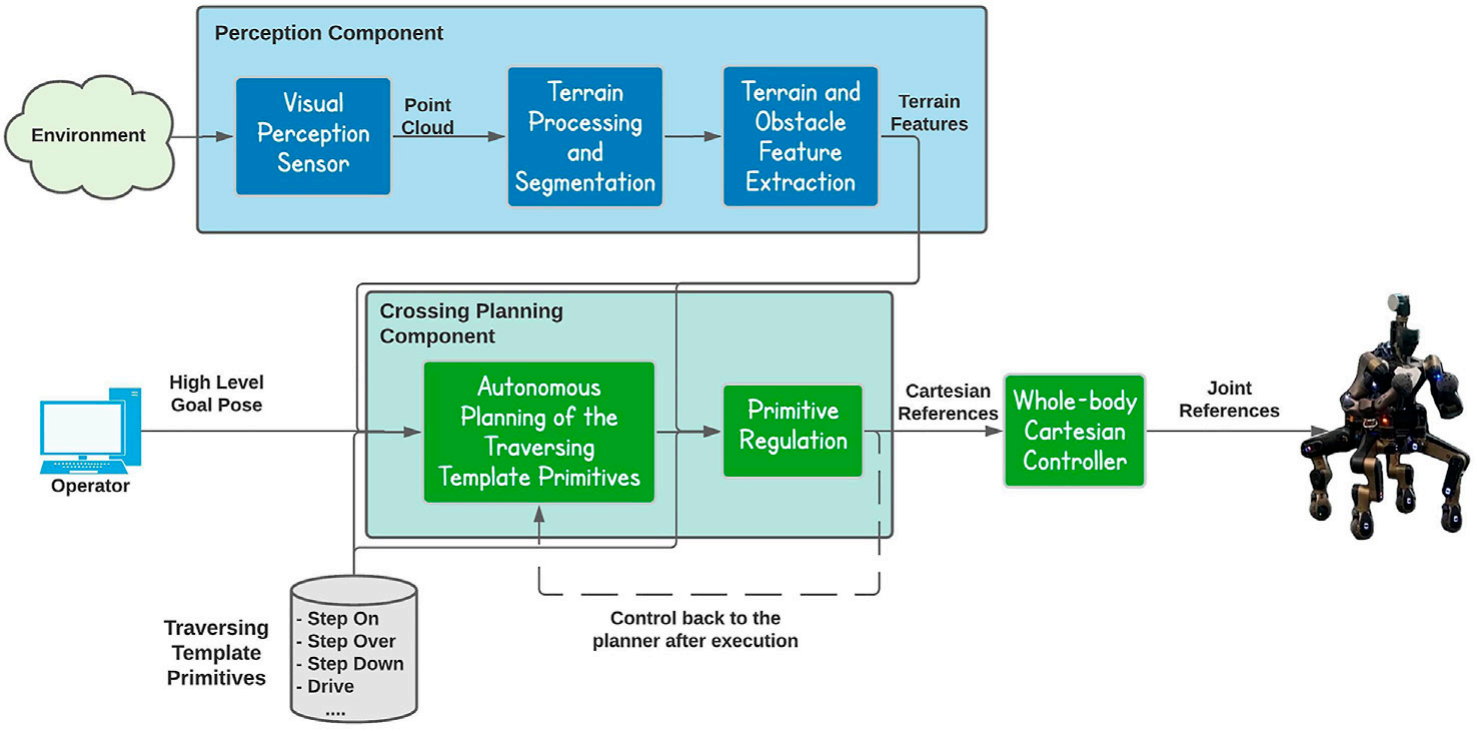
The proposed autonomous obstacle crossing framework composed by two core components, the Perception component and the Crossing Planning component.

An overview of the proposed autonomous obstacle crossing framework is introduced in (Figure 1) showing the core components of the framework and their interaction. The overall framework is composed by two core components. The first is dedicated to the terrain feature extraction and implements all the operations related to the perception data collection and processing, follow up by the terrain segmentation and feature extraction modules. The second component of the framework is responsible for the obstacle crossing planning and execution. It is composed by the planning module, which makes use of a set of traversing template primitives to plan and synthesize the traversing strategy. The selected traversing primitives are then adapted based on the properties of the terrain/obstacle features. Finally, the synthesized terrain traversing plan is executed by the whole body control module running on the CENTAURO platform.

### 3.1 Perception Component

**FIGURE 2:**
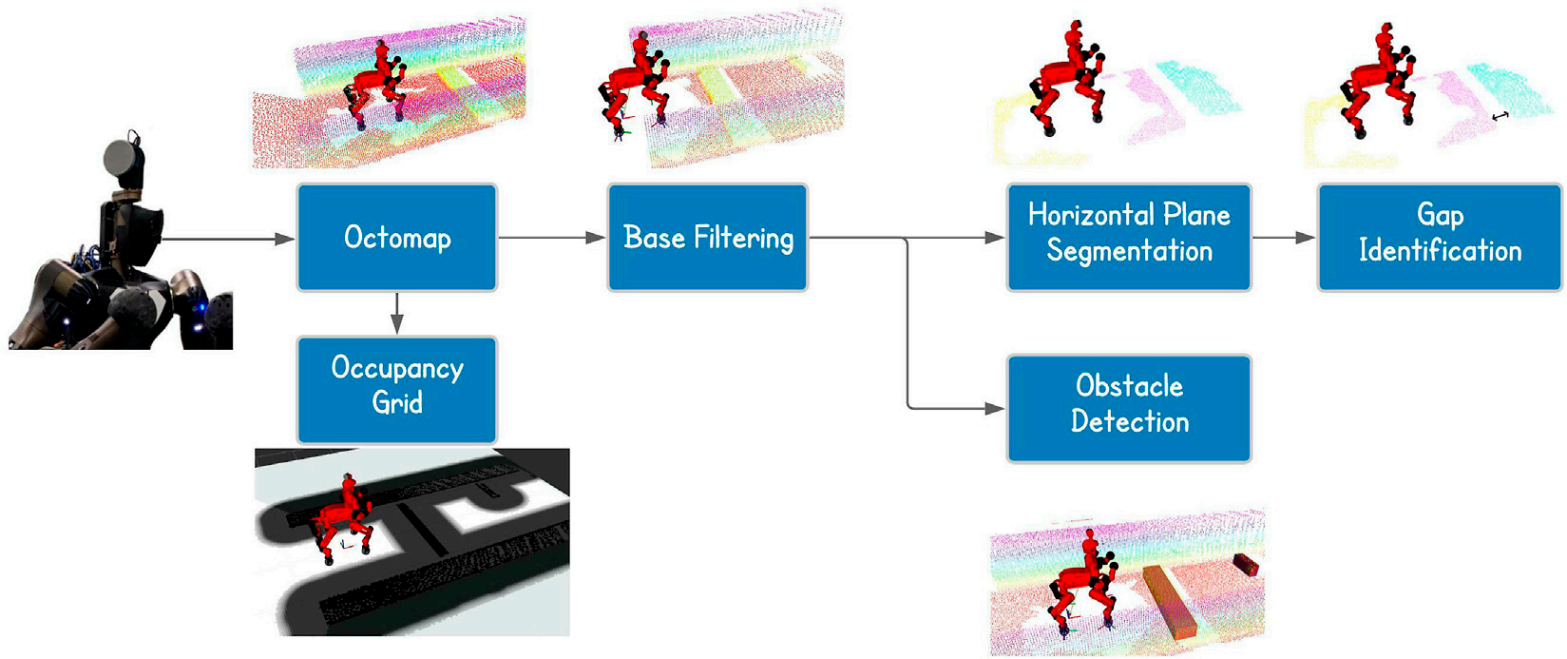
Overview of the pipeline of the perception component.

In this work a general representation of the environment is obtained using a rotating laser scanner sensor installed on the head of the CENTAURO platform. Starting from the point cloud data produced by the laser scanner sensor the goal of this component is to use these points to infer some kind of knowledge about the terrain features or obstacles that the robot will have to deal and interact with while performing the autonomous terrain traversing functionality. For this implementation we used the Point Cloud Library (Rusu and Cousins, 2011), which is a large scale, open source library for point cloud processing. An overview of the terrain feature extraction pipeline is introduced in Figure 2).

In the following we are going to present the perception pipeline defined even though it does not contain novel ideas, but it is useful to understand better which kind of information we extract from the scene. The pipeline process starts with an initial scanning phase which involves the execution of rotating actions with the laser scanner to obtain different scans of the environment around the robot. These scans are collected and merged by the octomap ROS node (Hornung et al., 2013), which allows to perform a probabilistic registration of the point clouds, obtaining in the end the map of the environment. Since the size of the map increases with the time passing, a filtering node is used to remove possible noise and keep only a portion of the space in front of the robot. This allows to reduce the number of points considered and so the computational time needed for further processing. The order of the filters applied took inspiration from Palnick (2014). In particular we used the following filters:

• *VoxelGrid Downsampling*: With this filter the entire 3D space is subdivided into voxels of the same dimension, where the voxel is the 3D equivalent of a grid cell. The points in each voxels are approximated with their centroid. This filtering technique allows to obtain a more homogeneous density in the point cloud. It is particularly useful with LiDARs since they provide an irregular density; much higher in the proximity of the sensor with respect to far areas;
• *Statistical Outlier Removal*: This filter considers, for each point, a small neighborhood to evaluate mean and standard deviation based on the distances between the points. If these values are outside a specified range, the point is considered as an outlier and so trimmed off the cloud. This filter is particularly useful for errors due to lights and reflection because they generally provide sparse outliers;
• *PassThrough Filter*: By using this filter we are able to keep or remove portion of a cloud based on the position along the three axis. We used this filter to consider only the points in front of the robot, trimming all the others.

Once that all the filters have been applied, the resulting output cloud is used to perform a horizontal plane segmentation, allowing us to obtain more information about the scene composition. In fact, the resulting planes can be used to identify possible gaps in the ground by looking at the distance between the planar regions found.

**FIGURE 3:**
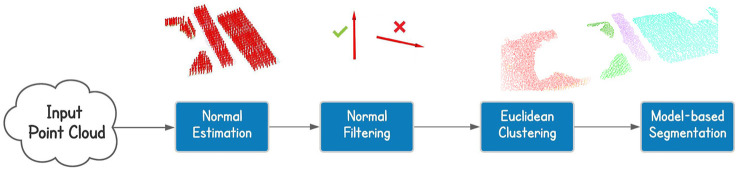
Cloud processing for horizontal plane segmentation. From the point cloud we perform normal estimation, normal filtering based on the orientation, euclidean clustering and model-based segmentation.

In parallel to the segmentation, the filtered cloud is also used to find obstacles. In particular, we firstly process the cloud with the purpose of keeping only the highest points for each x-y position, considering a discretization on the x-y plane with a resolution equals to the one of the sensor (3 cm). We then perform a Conditional Euclidean Clustering Extraction to split the resulting cloud into separated ones, based on the euclidean distance and the high of the points. On each of these clusters we fit a plane with a model-based segmentation algorithm. At this point we have the top surfaces of the objects and, to reduce the errors in the estimated yaw of the bounding box, we project all the points of the segmented plane on the x-y plane. This allows us to remove possible uncertainties due to double walls or remained outliers. Finally, we use the library function to obtain the oriented bounding box of the object perceived. In doing this we assumed to work with rectangular shapes for simplicity. Focusing more on the horizontal plane segmentation instead, we can summarize the pipeline and see the final and partial results in Figure 3.

At the beginning we estimate the normal vector for each point in the cloud. The normal estimation is obtained through a library function that computes the eigenvectors and estimates the normal. This library function is also provided for a GPU execution taking advantage of parallel computing, increasing the speed of the elaboration. Since CENTAURO is equipped with a GPU, we decided to use it for the normal estimation. The resulting data are used to filter the points based on the normal orientation, keeping only those points whose normal vector is at least inclined of 45 degrees with respect to the horizontal plane. We did not consider a more restrictive interval because the normal estimation is not perfect, especially with double walls, and otherwise, we could remove interesting points, affecting the accuracy of the overall procedure. At this point we can perform the actual segmentation, firstly computing the euclidean clustering, splitting the filtered point cloud into distinct ones, then, on each of these clusters we applied the model-based segmentation to extract the horizontal planes.

### 3.2 Crossing Planning Component

Once the terrain feature extraction module has been implemented, we leverage on the information extracted from the environment to autonomously overcome the perceived obstacles with the CENTAURO robot. The goal of this component is therefore to plan the trajectory that the end effectors (wheels) have to follow to cross the terrain features/obstacle that the robot is facing.

For the entire task we assumed to work in quasi-static assumptions to simplify the problem, maintaining the stability during the overall task keeping the Center of Mass inside the support polygon, which is defined by the wheels in contact with the ground during the stepping manoeuvre.

We decided to define a planning strategy based on a set of terrain traversing template primitives. In this implementation we considered the following primitives:

• *StepOn*: Performing a stepping action with one of the legs from a lower to a higher terrain height.
• *StepDown*: Performing a stepping action with one of the legs from a higher to a lower terrain height.
• *StepOver*: Performing a stepping action with one of the legs ensuring a height clearance over a certain obstacle height.
• *Drive*: Performing wheeled locomotion using the wheel end-effectors of the CENTAURO legs.
• *Reshape*: Performing a change in the shape of the support polygon by a rolling movement of the wheels.

**FIGURE 4:**
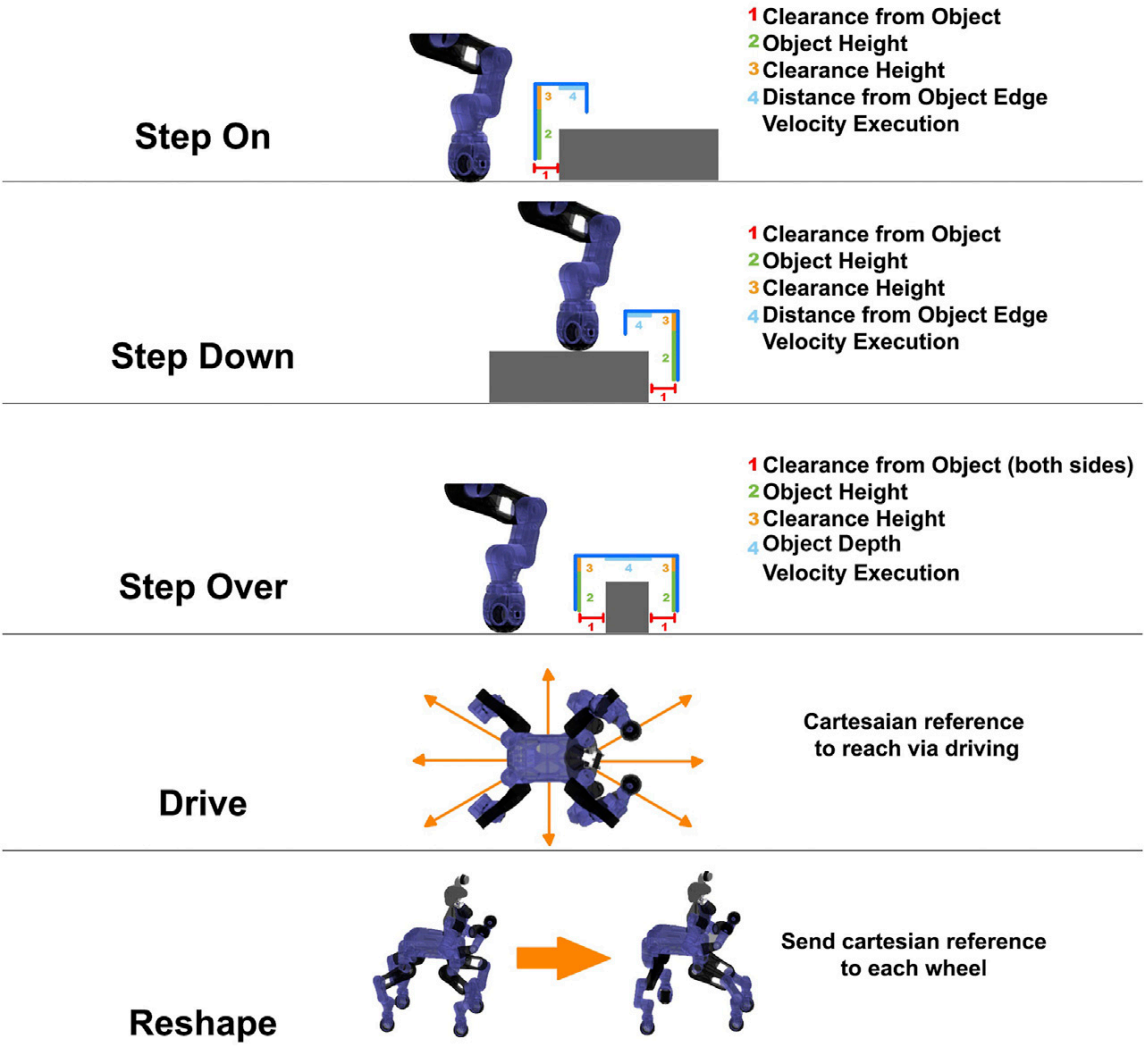
Graphical representation of the template primitives and their parameters. On the right side we can see the list of all the parameters that are considered in the definition of the primitive. In more details, for the stepping actions, the parameters allows to change at run-time the trajectory of the end effector based on the perceived obstacle.

The above template primitives have been parameterized with regards to the initial and final poses of the stepping/driving actions, the clearance height of the stepping actions as well as their executing velocity allowing to adapt to them accordingly to the needs of derived terrain traversing plan. A visual representation of the primitives and their adaptation parameters can be seen in Figure 4. In particular, the implemented primitives are autonomously executed in a sequential manner, based on the terrain features extracted, in order to build the terrain traversing plan that is sent to the controller of the robot. The composition of the terrain traversing plan based on the above mentioned primitives is the result of an offline planning that is performed in such a way that, at run-time, the traversing primitives can adapt their parameters (length of the stepping, the clearance height, etc.) based on the dimension of the object that the robot is approaching. In more details, the trajectory defined by each of the above primitives is not fixed in advance, but parametrized by a set of parameters that are regulated in an autonomous manner online to alter the form of the trajectory of the primitive and adapt it according to the properties of the perceived obstacles e.g., in order to avoid collisions with the obstacles. As an example, having the position of the object and its dimensions e.g., its height, the parameters are automatically selected to allow the robot to approach the object without touching and lifting the wheel at a suitable height to create the necessary clearance based on the elevation of the object. The depth dimension instead is used to define how much the wheel needs to be moved in order for the leg of the robot to reach the other side of the obstacle or its top surface, based on the selected primitive. In this work rectangular shaped objects are assumed and the primitives are modulated to negotiate this kind of terrain/obstacle shapes with the leg trajectory.

We chose to use a primitive-based hybrid planning because, compared to sampling-based methods, it is generally faster and easier to extend. In fact, we can easily remove or consider different set of defined actions, connecting them properly to achieve the result.

For the driving part we integrated in our module the ROS Navigation stack (Marder-Eppstein et al., 2010), which allows to perform 2D navigation decomposing the planning part into global and local. The global planner, implemented as an A* algorithm (Hart et al., 1968), allows to find the shortest path between the initial and ending point, taking into account the 2D occupancy grid and the inflation radius for the objects. This map allows to understand which are the areas traversable *via* driving. An example of the occupancy map is shown in Figure 2 where the black areas represent the obstacles, the gray the inflation radius from the obstacles and the white indicates the clear areas. The local planner instead is based on the Dynamic Window Approach (Fox et al., 1997), DWA, and tries to follow, as much as possible, the global plan, considering also the footprint of the robot to avoid collisions. The parameters of the planner have been tuned with several tests to allow correct execution of the path by our robot, considering a footprint that is slightly bigger than the actual to enhance robustness against uncertainty. The input taken by the navigation stack is the occupancy grid, provided by the octomap node, which provides information about the elements in the map, which are higher than the ground, since this considers a 2D motion only.

**FIGURE 5:**
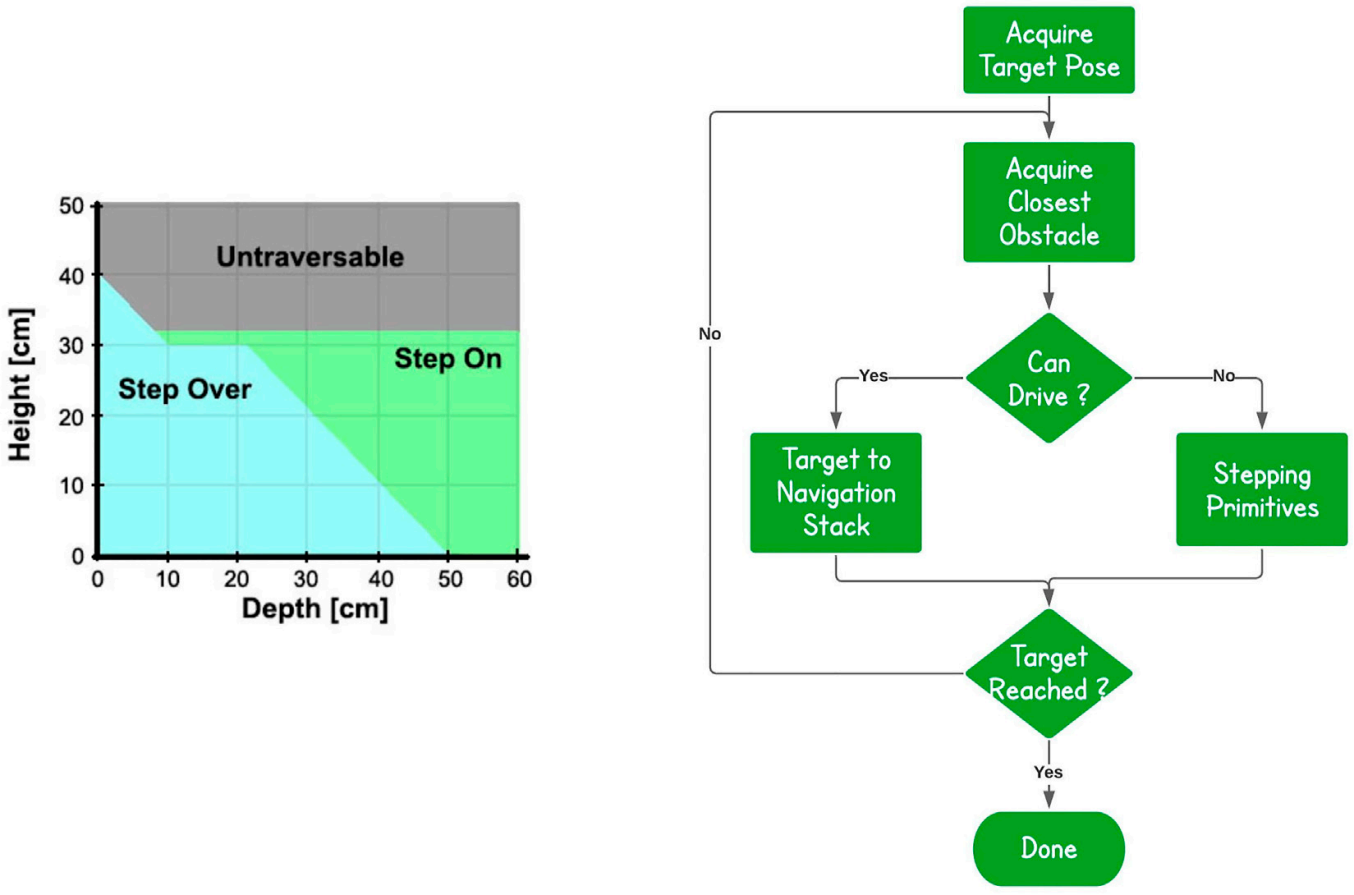
Primitive-based Reasoning. For each obstacle that the robot faces, we check if we can drive around it. If this is not possible the hybrid planner is used to derive appropriate traversing actions using the *StepOn*, *StepDown* and *StepOver primitives*.

The stepping part instead, as mentioned above, is achieved combining the available terrain traversing primitives with a finite state machine that selects the most appropriate primitive based on the terrain features extracted. In more details, the implemented reasoning is presented in Figure 5, where we arrive to the final target considering one obstacle at a time.

First of all, we acquire from the human operator the destination location goal on the ground that the robot shall reach. The information provided from the terrain feature extraction component is used to identify the closest obstacle to overcome on the way to the destination goal. The dimensional and geometrical properties of the object are evaluated to understand if the obstacle can be overcome or not by a driving primitive given the size of the CENTAURO wheel and its resulted capacity in overcoming obstacles up to certain height (within the range of 5 cm). Based on this assessment the stepping or the driving primitives are employed to negotiate the obstacle. This is iterated for each obstacle, until we arrive to the desired destination location in the space.

In more details, the reasoning applied to decide the traversing behaviour is based on a simplified kinematic of the robot’s leg and on several tests performed in simulation with obstacles of different dimensions. In particular, based on the leg design, the maximum elevation that we can reach is around 32 cm, except for thin obstacles since the wheel has to travel for a shorter distance. The stepping on procedures take into account mainly just the height of the obstacle, since the end point is based only on the edge. For the stepping over, instead, we need to cross with one action the entire obstacle, for this reason we cannot perform this action with objects that have a combination of height and depth over a certain threshold.

**FIGURE 6:**
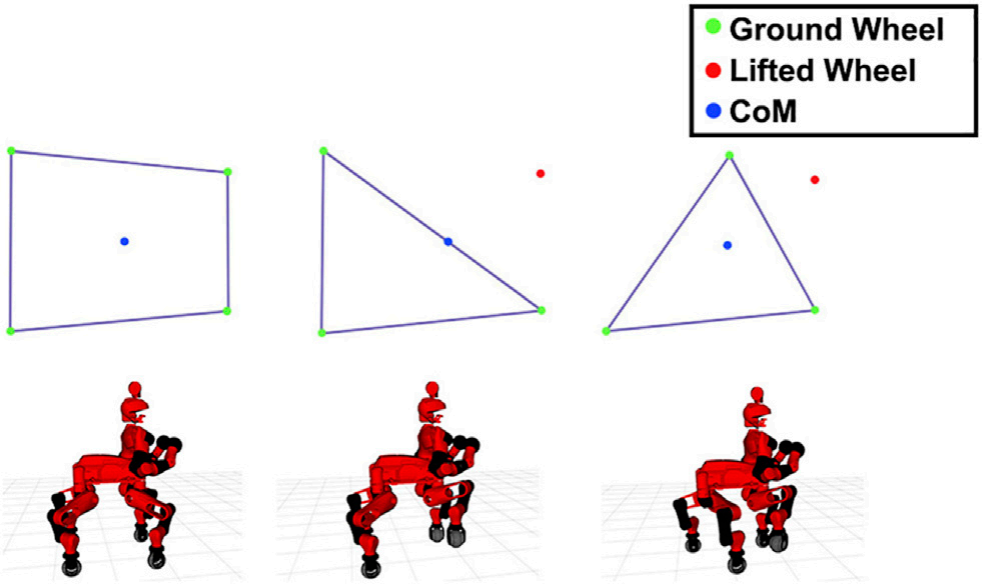
**(A)** Initial configuration, in which the wheels (green dots) are all in contact with the ground and the support polygon is a rectangle. **(B)** The result of lifting one wheel (red dot) without moving the others. Here the CoM (blue dot) is on the edge of the polygon. **(C)** The selected approach, where we move a wheel in order to obtain a more suitable support polygon with borderlines further than the location of the CoM.

In particular, for the stepping part, we start acquiring the information of the closest obstacle that is in front of the robot from the perception module. The planner, considering the terrain features, plans the sequential order of the primitives that has to be applied to deal with the obstacle and then it performs a regulation of the primitive parameters (stepping distance, clearance height, new foothold height) to adapt them to the geometrical and dimensional properties of the object to cross. At this point, the resulting references for the foot trajectories are sent to the robot controller to be executed by the robot. Following the execution of the sequence of primitives associated with the traverse of the obstacle, the planner continues with the generation of the next sequence of primitives to be executed based on the updated information provided by the perception module, concerning the follow up obstacle challenge to encounter. For the execution of the stepping primitives by each leg and in order to maximize the distance of the CoM from the borders of the support polygon, when the robot is standing on the three legs to execute the stepping action a primitive motion (*Reshape*) that performs a regulation of the support polygon shape is executed. In particular, at the beginning of each stepping action, the opposite wheel, with respect to the one that we are going to lift, is moved in order to align to the pelvis position so that the triangular support polygon is modulated to increase the distance of the CoM from that support polygon borderlines. The result of this can be seen in Figure 6.

Once that we increased the stability of the robot, we can lift the wheel of a quantity that is based on the height of the object. Then, if we are going to step over the object we move the wheel in such a way to arrive on the other side of the obstacle, otherwise we move to the beginning of the obstacle plus a threshold, in order to avoid to place the foot on the edge. Finally we lift down the wheel and move back the opposite wheel, we moved for stability, to its original position.

## 4 Results and Discussion

### 4.1 System Overview

**FIGURE 7:**
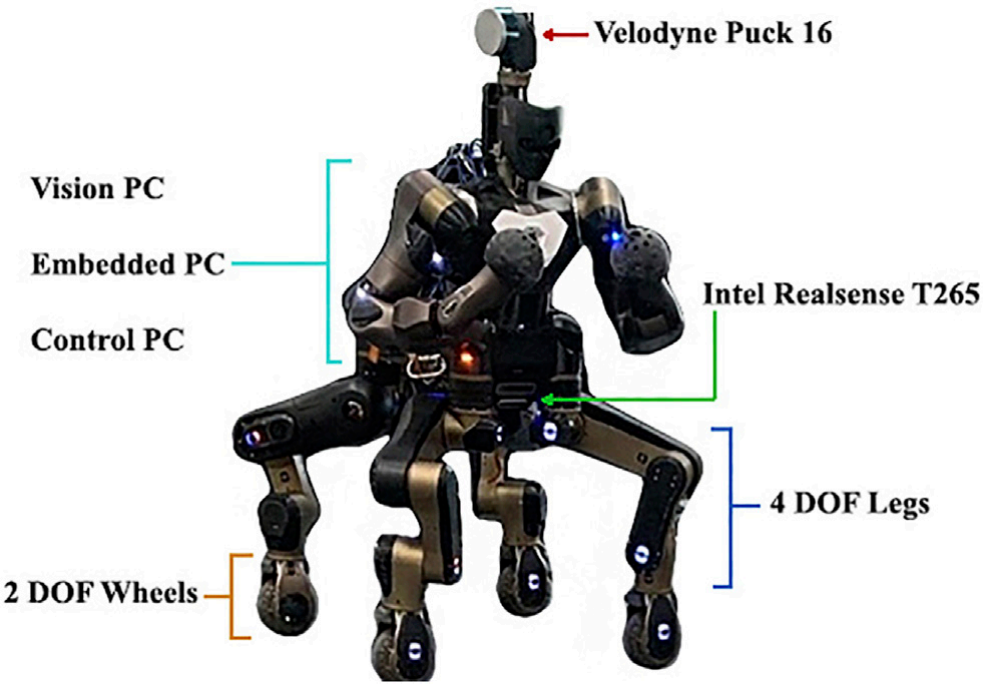
The CENTAURO hybrid legged/wheeled robotic platform with indications of its mobility system, perception and on board computational components.

The evaluation of the proposed terrain traversing framework was performed on the CENTAURO robot (Figure 7) which is a centaur-like robot with four articulated legs and a humanoid upper body. From a locomotion point of view CENTAURO was designed to be hybrid. This was chosen to overcome the limits of wheeled and tracked robots, that can drive only on flat terrains. In fact, CENTAURO has four 5 DoFs legs, ending in 360^o^ actuated steerable wheels. At the same time its anthropomorphic upper body endows the robot with enhanced manipulation capabilities making CENTAURO an excellent mobile manipulation platform that can be used to address in a range of applications.

Analyzing its sensory system, CENTAURO is equipped with a Velodyne Puck 16 sensor (LiDAR), which is a continuously 3D rotating laser scanner that provides range measurements. This sensor allows to obtain a 3D representation of the environment as a point cloud and is mostly used in this work. In addition to the Velodyne, the robot is equipped with an Intel Realsense T265 camera^2^ that assists in the localization of the robot within its environment. This device has an IMU and two greyscale fish-eye lenses that are used to estimate the pose of the sensor. We used this data to correct the position of the robot, being able to localize it, while building the map.

The onboard software and control architecture of the robot employs the following main elements: XBotCore (Muratore et al., 2017), OpenSoT (Hoffman et al., 2017) and CartesIO (Laurenzi et al., 2019).

The first one is an open-source, light-weight platform for robotics system that was designed to be both a RT robot control framework and a software middleware. It provides a simple and easy-to-use middleware API for both RT and non-RT control frameworks. OpenSoT is an open-source software library developed to address whole body motion generation and control of redundant robots. It includes high-level interfaces to the state-of-the-art algorithms used for kinematic and dynamic modelling, quadratic programming optimization, cost functions and constraints specification. Finally CartesIO extends OpenSOT with additional layers that permit the user to define in an intuitive manner based on a set of tasks a Cartesian controller without the need of writing code. To achieve this, the team designed an auto-generated interface to send commands to the Cartesian controllers using ROS (Quigley et al., 2009).

In terms of onboard computational resources, CENTAURO is equipped with three different PCs. We used the *EmbeddedPC* to run XBotCore, the *ControlPC* for the Cartesian Controller and finally the *VisionPC* for the architecture proposed in this manuscript.

The first set of experiments targeted to test and evaluate the perception module. In particular we assessed the accuracy of the obstacle detection running several tests with different rectangular objects.

**FIGURE 8:**
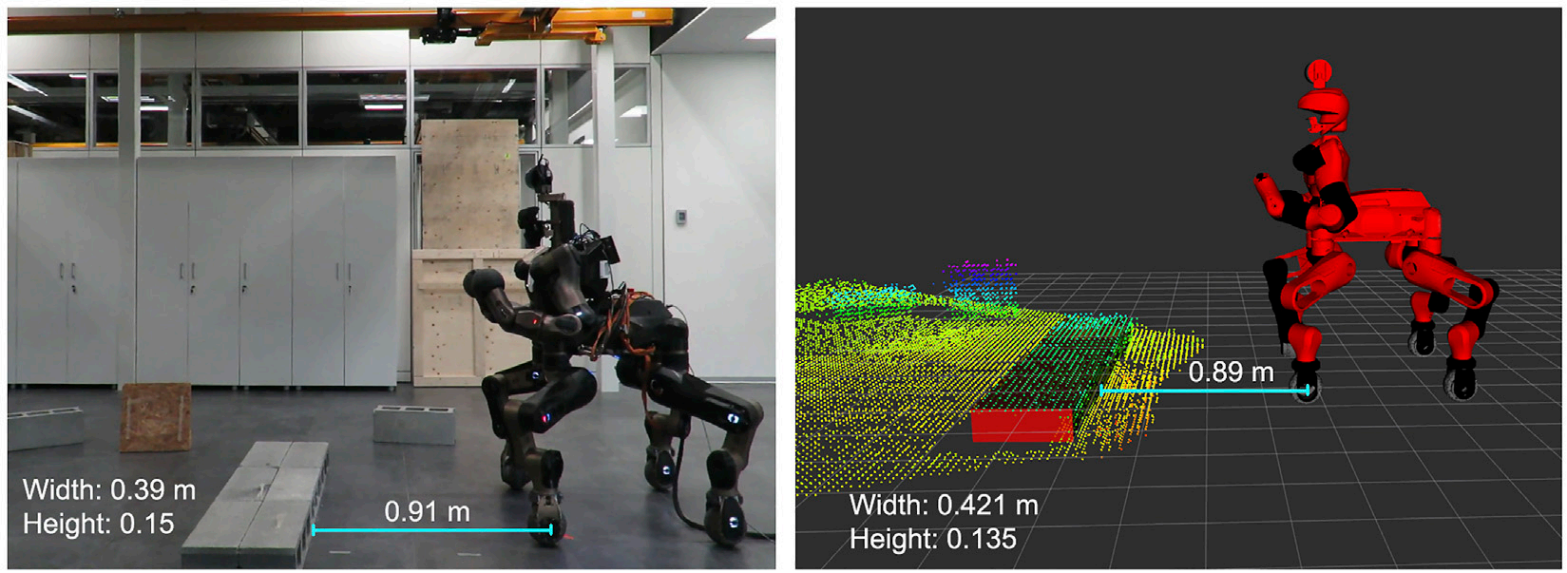
Comparison of the real world scenario and the perceived one. The error in both dimensions and distance between wheels and object is smaller than the sensor resolution, 3 cm.

We compared the actual position and dimensions of the obstacle with the estimated ones. For the height of the obstacle we obtained generally more accurate results, since we consider an average of the z values of all the points of the top surface, otherwise, keeping just the highest one may result in an outlier. In this way we obtained an error that is less than 2 cm. The other two dimensions of the obstacle are extracted from the bounding box and resulted in errors smaller than 4–5 cm, while the error of the center of the object was within the 1 cm. The achieved accuracy results are considered reasonable given the 3 cm resolution of the sensor used, therefore the expected error was 6 cm, 3 cm per each side. Further improvement of this accuracy would require the use of a more accurate sensor. In Figure 8 we can see the point cloud obtained in the real case scenario, with the oriented bounding box estimated from it and the measures considered for the evaluation. In Table 1 are specified the quantitative results of the perception module, evaluating the errors of both depth and height estimation obtained in simulation environment and also on the real robot. These measurements were extracted from the Obstacle Segmentation module running at 10 Hz considering objects of different dimensions, in more details: height from 0.07 to 0.35 cm and width from 0.10 to 1.60 cm. Moreover we considered also objects at different distance and orientation with respect to the actual robot position.

**TABLE 1:** Statistical results of the estimate of the obstacle dimensions in both simulation and on the real robot. The data were extracted considering rectangular obstacles of different dimensions, placed at different distance and orientation with respect to the robot.

	Simulation results	Real robot results
Min. Depth Error	0.007 m	0.007 m
Max. Depth Error	0.053 m	0.045 m
Avg. Depth Error	0.026 m	0.026 m
Std. Dev. Depth	0.0302 m	0.0297 m
Min. Height Error	0.001 m	0.004 m
Min. Height Error	0.010 m	0.020 m
Avg. Height Error	0.005 m	0.006 m
Std. Dev. Height	0.0051 m	0.0062 m

### 4.2 Scenarios Considered

To run and validate the primitives and the autonomous hybrid navigation planner, we then executed trials considering different obstacles, at the beginning separately, then sequentially to build a more complex and challenging environment. In more details we considered the following four obstacle sizes:

• Obstacle-A: Rectangle of 0.20 × 1 × 0.15: Step over;
• Obstacle-B: Rectangle of 0.40 × 1 × 0.15: Step on;
• Obstacle-C: Rectangle of 1.50 × 0.8 × 0.15: Step on;
• Obstacle-D: Rectangle of 0.15 × 0.45 × 0.19: Drive around.

**FIGURE 9:**
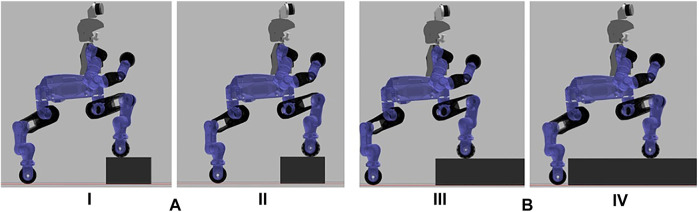
Difference in the stepping on approach after stepping on with the front wheels: **(A)** Front wheels are much closer to the end of the object rather than the rear wheels with the beginning of the obstacle. The robot steps on with the front wheels (I) and drive until the front wheels reach the end of the obstacle (II). At this point the robot will step down with the front wheels before considering the stepping on with the rear ones. **(B)** The opposite case, in which the robot has to step on with the front wheels (III) and drive until the rear wheels will be close to the object (IV). At this point the robot will step on also with the rear wheels.

The selected obstacles allowed us to verify all possible crossing approaches achieved through the combination of the motion primitives defined. The first obstacle requests a stepping over primitive, the second and the third allow to test two different behaviours of the stepping on procedure, while the last obstacle involves navigation to drive around the object. In particular, in the second case we have to cross the obstacle stepping on with the front wheels and then stepping down, arriving in a configuration in which the obstacle is between the front and the rear wheels. While in the other case we have to step on the obstacle with all the wheels in order to cross it, as visualized in Figure 9. The choice is based on the dimension of the obstacle and so the distance between the front wheels and the end of the object on which they are and between the rear wheels and the beginning of the obstacle that they still have to approach.

**FIGURE 10:**
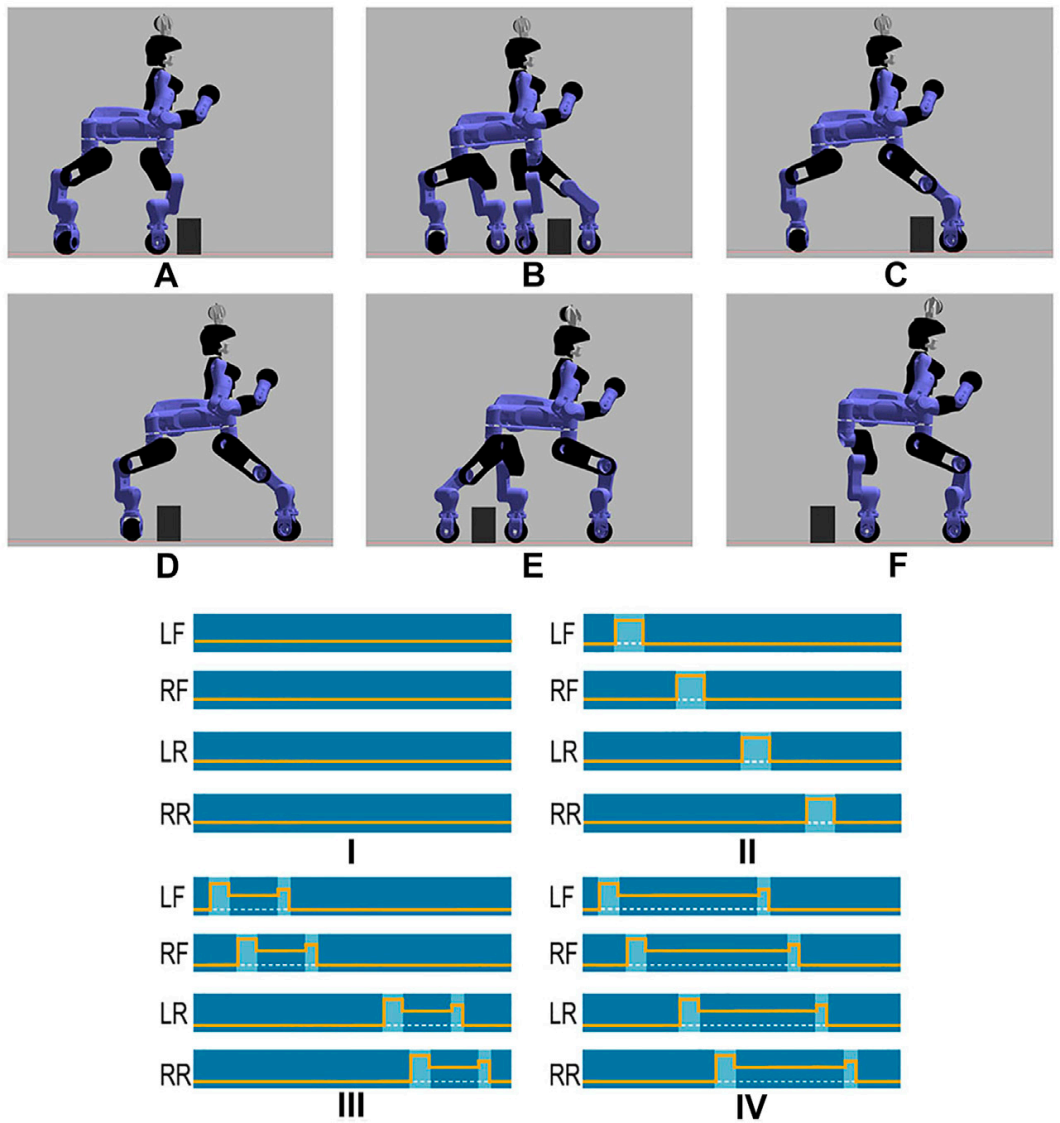
Sequence of the stepping over strategy applied in simulation environment with an Obstacle-A. **(A)** We approached the obstacle, stopping at a safety distance from it, **(B)** step over with the left front wheel, **(C)** step over with the right front wheel, **(D)** drive, stopping with the rear wheels close to the object, **(E)** step over with the left rear wheel, **(F)** step over with the right rear wheel, completing the task. In the bottom you can see the motion modalities considered in the framework. Dark blue color indicates that the corresponding wheel is in contact with the ground; the orange line instead is the Z value of the wheel, while the dashed white one shows the terrain level. (I) Driving on flat surface, (II) Traversing Over an obstacle modality making use of sequential stepping over primitives on the individual legs, (III) Traversing modality for a small depth obstacle making use of stepping on and stepping down primitives executed by the four legs, (IV) Traversing modality for a large depth obstacle making use of stepping on and stepping down primitives executed by the four legs.

Before executing a trial with the real CENTAURO robot a trial was first executed in Gazebo simulation environment to verify the correct operation. The top images of Figure 10 introduce a sequence showing the stepping over approach of an Obstacle-A. While the bottom images describe the motion modalities that are considered inside the planner for the experiments carried out.

**FIGURE 11:**
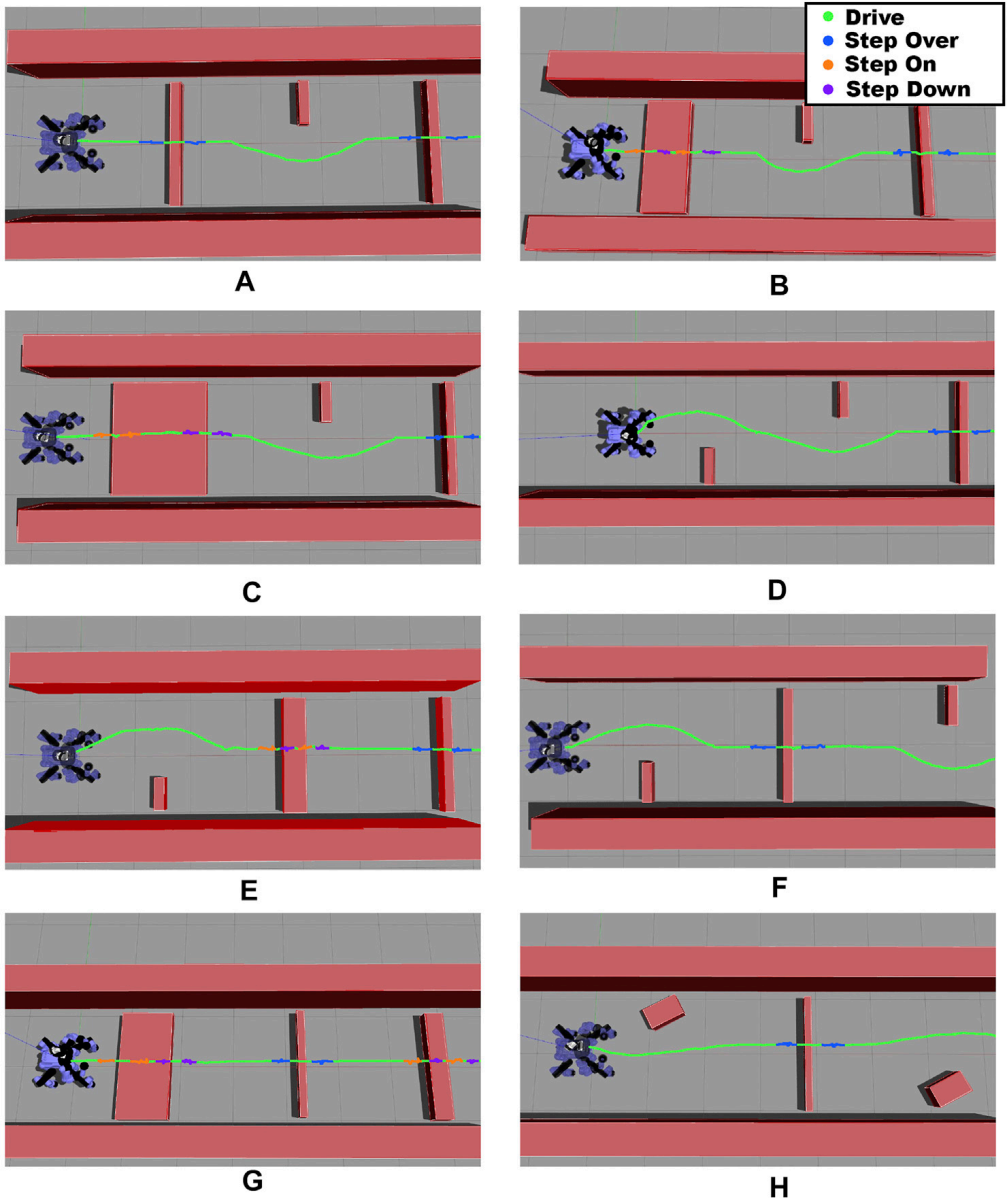
Scenarios considered in simulation to check the robustness of the implemented framework. The colored line represents the trajectory followed by the pelvis during the overall task and it is mapped as follows: green for driving, blue for stepping over, orange for stepping on and purple for stepping down.

Furthermore, in simulation environment we performed additional tests to evaluate the robustness of our approach in a number of different obstacle configurations. In particular, we considered eight scenarios with a different arrangement of obstacles, as it can be seen in Figure 11. For each of these scenarios we run 5 trials changing the initial position of the robot along the *x* and *y* axis in a neighborhood of 0.7 m from the zero position (the actual position of the robot in the image). In all the trials performed the proposed method generates a correct and effective plan to cross the obstacles by combining the available primitives. As an example, considering the scenarios F and H, the plan starts selecting the driving primitive to avoid the obstacle on the side of the corridor, then the step over is used to overcome the small obstacle that blocks the path and finally driving again to reach the target location assigned. In the scenario B instead the plan begins with stepping on and stepping down for the front wheels, then the same primitives are selected for the rear ones. At this point the robot avoids the obstacle via driving arriving in front of the last obstacle that is crossed using the stepping over primitive.

**FIGURE 12:**
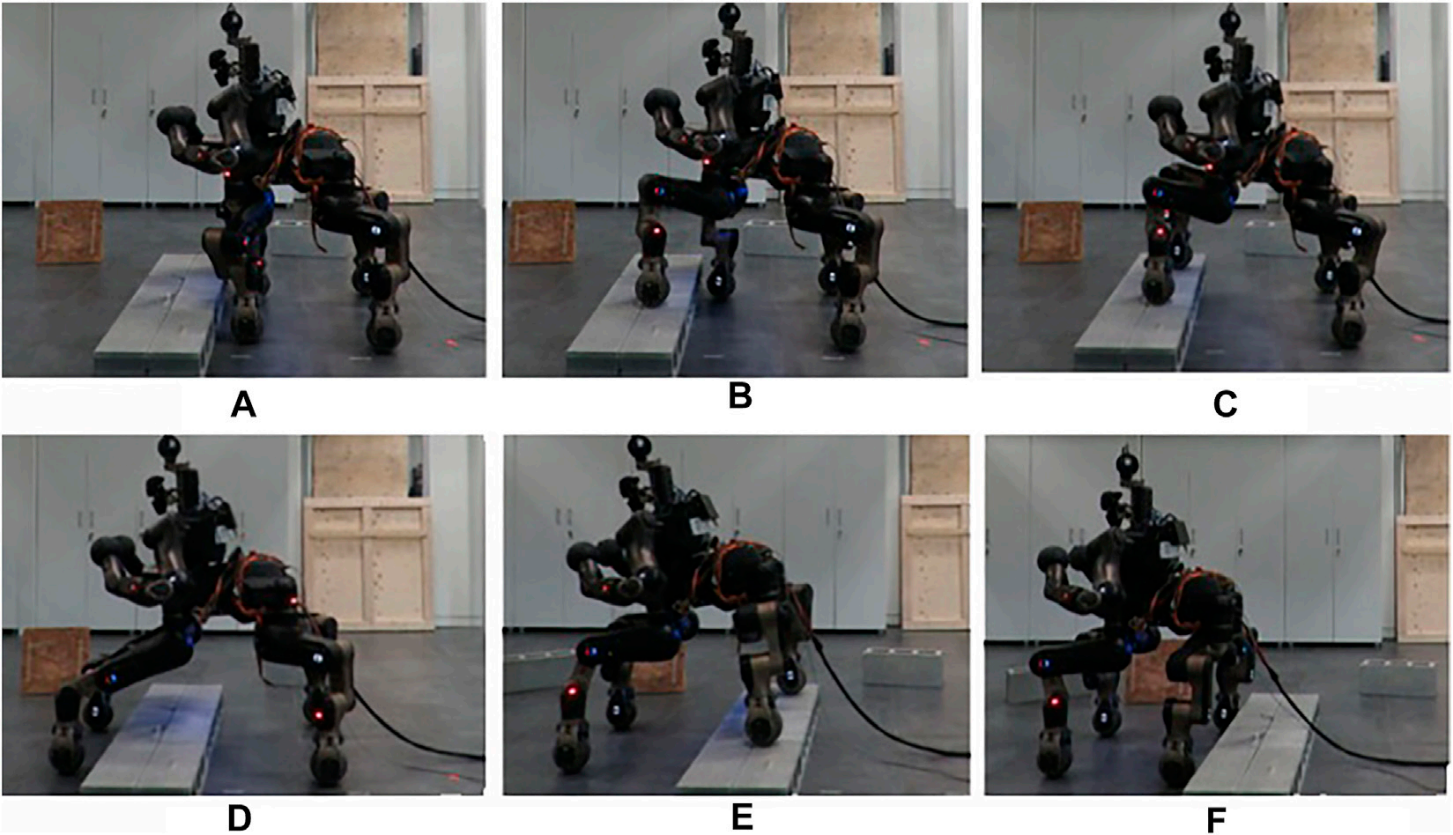
Sequence of the stepping on strategy applied on the real robot with an Obstacle-B. **(A)** The robot is close to the obstacle, **(B)** step on with the front wheel, **(C)** step on with the front wheels, **(D)** step down with the front wheels, **(E)** step on with rear wheels, **(F)** robot completed the task, crossing the obstacle.

Once the trials were verified in simulation, equivalent trials were carried out on the real CENTAURO robot considering, also at this stage, we started with one obstacle at a time. In all the scenarios that were executed with the real robot, the estimation error on the dimensions of the obstacle was lower that 3 cm, per each side, while the robot successfully overcome the provided obstacles without negotiating its balance while performing the crossing primitives. During the experiments the robot operated without a gantry system while being tethered for power and communication. These tests permitted us to validate the template primitives and the trajectories specified for the feet. A stepping action executed by the robot during this trial can be seen in Figure 12.

Up to now, we checked the correctness of the execution on single obstacles. The next experiment targeted to evaluate the proposed method while the robot has to traverse a series of different obstacles arranged one after the other and separated by approximately 1.8 m from each other. At the beginning of the experiment, we acquire a preliminary view of the environment with the LiDAR, building the map and the occupancy grid. Then, after having crossed each obstacle, we reset the perception and rotate the robot to acquire more information for the size and position of the next obstacle.

**FIGURE 13:**
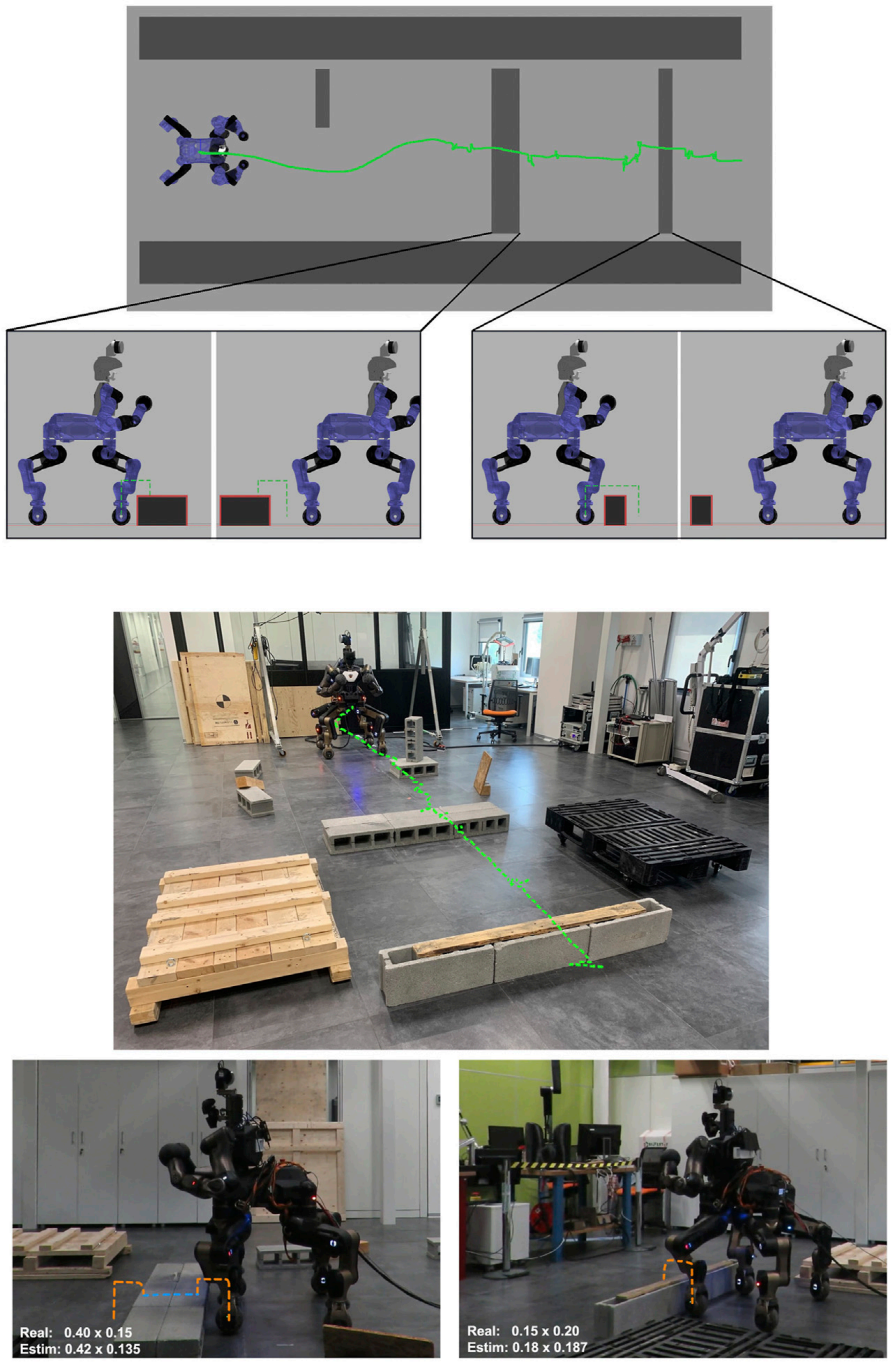
Final experiment in simulation and in real world case. The top depicts the scenario considered with the pelvis trajectory shown in green. The side views demonstrate the stepping actions, where the orange dashed line shows the trajectory followed by the wheel during the lifted execution while the blue one shows the trajectory followed using the driving primitive. The first obstacle is avoided driving around, the second one stepping on and down, finally the last one is crossed stepping over.

**FIGURE 14:**
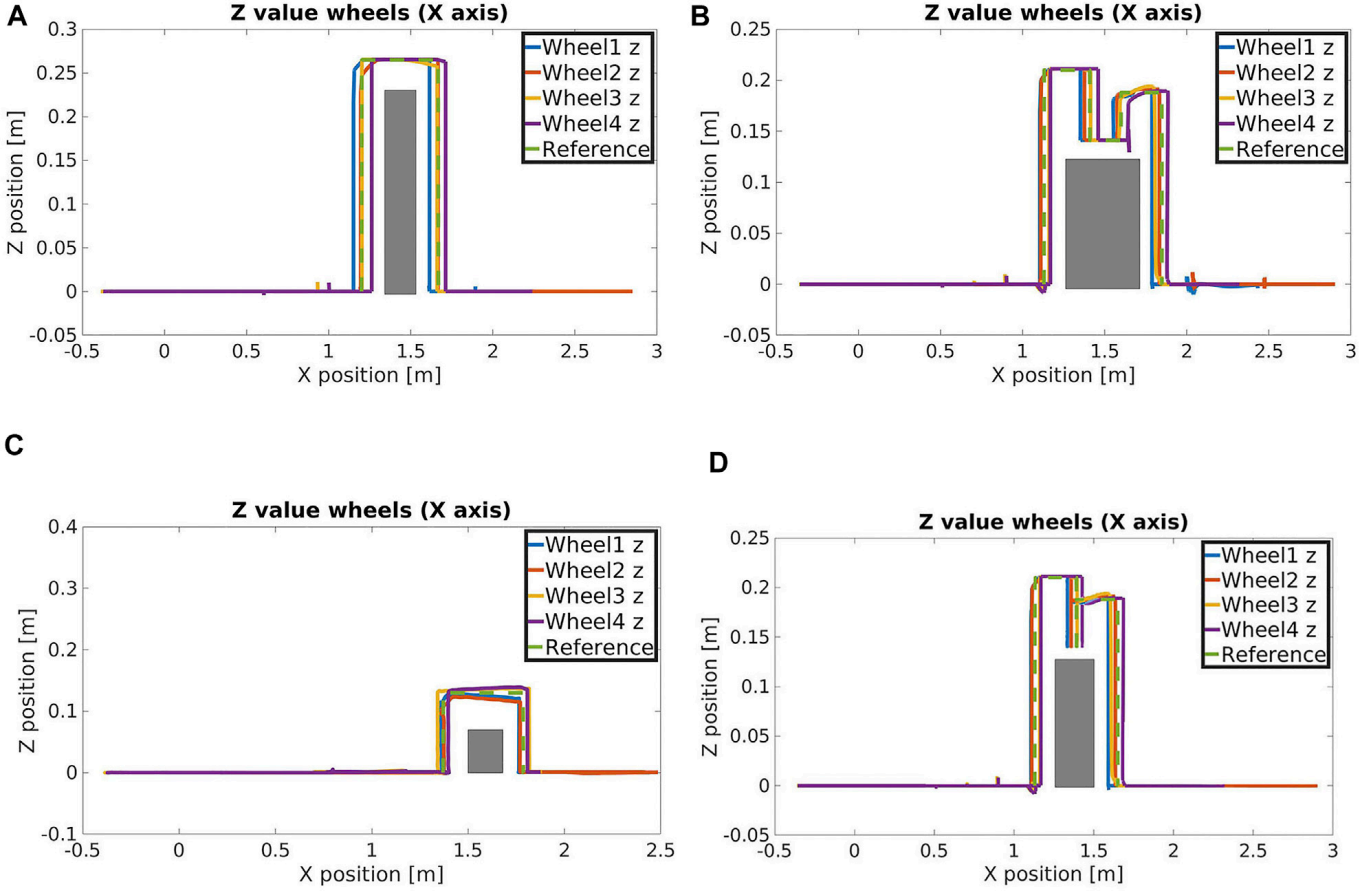
Z position of the wheels during **(A)** stepping over, **(B)** stepping on, **(C)** stepping over 7 cm height obstacle and **(D)** crossing a small obstacle without stepping over primitive.

Figure 13 introduces the overall scenario, in simulation and in the real world, where the first obstacle can be crossed by driving around, using the ROS navigation stack. At this point, the robot arrives in front of the second obstacle facing it. After the extraction of the features from the perception module, the planner selects autonomously the step on strategy and starts approaching the object. Using the perception system the desired distance from the object is evaluated and used to regulate the robot motion until the robot arrives in the right pose/distance in front of the obstacle. The planner uses the desired stepping primitive, adjusting the parameters based on the dimension of the obstacle. A similar approach is followed for the last obstacle, that since has dimensions under a certain width threshold, it can be traversed *via* a stepping over primitive. Figures 14A,B present the position of the wheels during the two stepping tasks and the desired reference that they should follow. We can see that not all the wheels starts to lift at the same distance, this is related to a not perfect alignment of the robot in front of the obstacle. Despite this, thanks to the tuning of the thresholds and parameters on the real robot, the algorithm is robust enough to allow the correct execution of the task even with an error in the alignment. In Figure 14B we can also notice that the elevation reached by the wheels during stepping on and stepping down is different. This is due to the fact that for the stepping on we may have at maximum 3 cm of error in the estimation (the resolution of the sensor used). For this reason we decided to increase slightly the maximum elevation reached to 7 cm over the estimated height to improve the safety. For the stepping down instead we do not have this problem, as we only need to lift the wheel from the surface of the obstacle and we can use a smaller clearance.

The overall experiment was carried out in 9 min on the real robot and in less than 7.5 min in simulation. This difference was due to some limitations we encountered using the real sensors. In fact, the Realsense T265 camera sometimes disconnected and the Velodyne sensor had to remain fixed, forcing us to rotate the robot instead of the LiDAR to acquire a representation of the environment.

In terms of performances, the time used for the planning is in the order of 1 ms, since it works as a state machine so, once the obstacle dimensions are extracted, the algorithm decides immediately the most suitable crossing behaviour. The workflow is quite straight-forward and the adjustment of the primitives requires a negligible amount of time. On the real robotic platform we made tests with single obstacles, being able to complete the task 2 times over 3 (for the stepping obstacles), 3 times over 3 with the driving and avoidance. For what concerns the full experiment, the one with all the three obstacles in a row, we made only one test and it was successful.

In addition, to further demonstrate the adaption of the template motion primitives, we present in Figure 14C the trajectory followed by the wheels of the robot while performing a stepping over task with an obstacle of 7 cm height. As we can see, the automatic adjustments of the parameters allows the wheels to lift of a quantity that depends on the obstacle height.

Finally we made an additional experiment to show the adaption of the planner in case of a primitive removal. In more details, we considered the same scenario of the step over task, with an Obstacle-A, but we disabled the step over primitive. In this way the planner has to use the remained primitives (drive, reshape, step on, step down) to find a different plan to accomplish the task.

In the resulting planned trajectories, as shown in Figure14D, the robot crosses the obstacle using stepping on and stepping down primitives on their edge limits since the length of the top surface of the obstacle is very similar to the diameter of the wheels. For this reason there is no driving part between the stepping on and stepping down, differently from what we saw in Figure14B.

With these tests we were able to validate our terrain traversing template primitives and their automatically online adjustment based on the obstacle considered, being able to demonstrate also the autonomous capabilities of the CENTAURO robot with a hybrid primitive based planner driven by the perception. The robot was able to identify the location and the size of the obstacles, one after the other, negotiating different strategies to cross them, arriving in an autonomous manner, to the target location assigned. In the real case scenario we had few limitations related to the 3D LiDAR that forced us to re-acquire the environment before approaching every obstacle, but this can be easily automated in order to have the operator only for monitoring and providing the high level goal.

## 5 Conclusion

In this work we introduced an autonomous hybrid locomotion planner based on predefined set of motion primitives. The proposed planner was validated on the legged-wheeled robot CENTAURO enabling the robot to execute autonomously traversing actions on obstacles identified by the robot perception system. The planner autonomously selects the suitable crossing action from a set of motion primitives based on the dimensions of the obstacle to negotiate. The proposed method allowed us to start exploiting the autonomy of the CENTAURO robot thanks to the introduction of the perception in the overall pipeline. We are currently working on extending the architecture with terrain traversability features that abstract from the shapes of the objects and improve the planning part by introducing new actions and higher level of reasoning. This will extend the capabilities of CENTAURO permitting the robot to adapt autonomously to more irregular shape obstacles and real-case terrain geometries and features.

## Data Availability

The original contributions presented in the study are included in the article/Supplementary Material, further inquiries can be directed to the corresponding author.

## Footnotes

1
https://www.bostondynamics.com/spot

2
https://www.intelrealsense.com/tracking-camera-t265/
